# Addiction and pregnancy: case report on gender perspective.

**DOI:** 10.1192/j.eurpsy.2024.831

**Published:** 2024-08-27

**Authors:** L. Bueno Sanya, O. De Juan Viladegut, L. Olivier Mayorga, H. Andreu Gracia, P. Barrio Giménez

**Affiliations:** Psychiatry and psychology, Hospital Clínic de Barcelona, Barcelona, Spain

## Abstract

**Introduction:**

In recent years, interest has grown in understanding the particularities of addiction in women. One of these singularities, with an important impact on public health, is pregnancy. Substance use during pregnancy has increased in recent decades. Given that addiction is mainly a chronic disease of the brain circuits of reward, motivation and memory, an event such as pregnancy does not exempt people who suffer from substance use disorder (SUD) from the difficulties of achieving abstinence. Moreover, as addiction often involves cycles of relapse and remission, pregnant women can also suffer a relapse even if they previously had achieved abstinence. On the other hand, given that addiction is a disease with a significant social component, we can find patients in precarious economic and social situations who suffer unwanted and therefore unplanned pregnancies.

**Objectives:**

To describe the case of a pregnant woman with SUD for multiple drugs who is admitted to the inpatient ward of the Hospital Clínic of Barcelona for detoxification. Also to reflect, taking in to account gender perspective, on the particularities of substance use in women on childbearing age.

**Methods:**

We present the case of a 25-year-old woman, six weeks pregnant and homeless, who was admitted to the inpatient ward for presumed psychotic symptoms. Even if the patient had a history of intravenous heroin, cocaine, and methamphetamine use, during the admission she only admitted current alcohol consumption (3 UBE/day). During admission, the patient decided to undergo a legal voluntary abortion. Regarding this case, we did a literature review on the consequences of different substances use to pregnancy (to both the fetus and the mother’s health). Likewise, we reflected on interventions that could be carried out in community mental health facilities to detect cases like the one exposed and provide help.

**Results:**

Poor obstetric outcomes are six times higher in patients who use substances. Newborns can have withdrawal syndrome, spontaneous abortion, pre-term birth, fetal malformations and fetal growth restriction. More than 50% of women of reproductive age use drugs, mainly alcohol. Routine screening and education of women of reproductive age is the best way to reduce substance use during pregnancy.

**Conclusions:**

We consider it essential for a better management of these patients to remember that the evidence suggests that the decisions that addicts make are mainly driven by a pathology, not by a moral failure. However, pregnancy can be a moment of greater motivation to stop using drugs, given that it is a high risk situation for the mother and the fetus.

**Disclosure of Interest:**

None Declared

